# Harnessing the Electronic Health Care Record to Optimize Patient Safety in Primary Care: Framework for Evaluating e–Safety-Netting Tools

**DOI:** 10.2196/35726

**Published:** 2022-08-01

**Authors:** Georgia Bell Black, Afsana Bhuiya, Claire Friedemann Smith, Yasemin Hirst, Brian David Nicholson

**Affiliations:** 1 Department of Applied Health Research University College London London United Kingdom; 2 North Central London Cancer Alliance London United Kingdom; 3 Nuffield Department of Primary Care Health Sciences University of Oxford Oxford United Kingdom

**Keywords:** primary care, patient safety, electronic health record, safety, optimize, framework, evaluation, tool, diagnostic, uncertainty, management, netting, software, criteria

## Abstract

The management of diagnostic uncertainty is part of every primary care physician’s role. e–Safety-netting tools help health care professionals to manage diagnostic uncertainty. Using software in addition to verbal or paper based safety-netting methods could make diagnostic delays and errors less likely. There are an increasing number of software products that have been identified as e–safety-netting tools, particularly since the start of the COVID-19 pandemic. e–Safety-netting tools can have a variety of functions, such as sending clinician alerts, facilitating administrative tasking, providing decision support, and sending reminder text messages to patients. However, these tools have not been evaluated by using robust research designs for patient safety interventions. We present an emergent framework of criteria for effective e–safety-netting tools that can be used to support the development of software. The framework is based on validated frameworks for electronic health record development and patient safety. There are currently no tools available that meet all of the criteria in the framework. We hope that the framework will stimulate clinical and public conversations about e–safety-netting tools. In the future, a validated framework would drive audits and improvements. We outline key areas for future research both in primary care and within integrated care systems.

## Introduction

*Safety-netting* was first formally defined in the mid-1980s by Neighbour [1] and has since come to be viewed as a best practice for managing diagnostic uncertainty [2]. This is particularly relevant to primary care, wherein clinicians hold responsibility for weighing up the costs, risks, and benefits of monitoring symptoms against those of ordering tests, investigations, and referrals for further care. Safety-netting includes verbally advising to patients to practice self-care, monitor symptoms, or seek further advice if their symptoms have not resolved. Safety-netting is part of many primary care presentations, given the high volume of patients with undifferentiated nonspecific symptoms. For these patients, serious disease is a rare but important component of a differential diagnosis [3,4].

Several studies have highlighted the importance of recording safety-netting advice in patient records [5-7]. Examples of such advice include ensuring that at-risk patients are monitored, providing a reminder of the advice, facilitating the continuity of care, and maintaining a medical-legal record. Despite their importance, safety-netting advice is not often recorded in medical notes [8]. There have been calls to improve the recording of safety-netting to facilitate follow-up and monitoring. More recently, commercial e–safety-netting tools have been developed to assist health care professionals in managing diagnostic uncertainty [9-11]. These tools may be integrated within the electronic health record (EHR) or provided by a third-party application.

The aim of this paper is to consider how e–safety-netting tools need to be developed in order to improve diagnostic safety in primary care. We also outline an emergent framework of criteria for e–safety-netting tools that can be used to facilitate evaluation and outcome measurement [12].

## Safety and Safety-Netting in Primary Care

The management of diagnostic uncertainty in primary care is a part of every primary care physician’s role [13]. Safety-netting mitigates the risks associated with some techniques, thereby allowing physicians to manage diagnostic uncertainty. For example, the safe use of the “test of time” allows for the expected progression of a primary care physician’s initial diagnosis to be observed. Safety-netting increases safety by providing patients with information about concerning symptoms and what to do if they arise [8-10,14]. Signposting to other sources of information or to other services (eg, out-of-hours services) is also a common component of safety-netting [10]. Effective safety-netting is important, since it can have implications for a patient’s outcomes by preventing misdiagnoses, complications, and delayed referrals [3,15]. It may also have workload implications by safely reducing the number of unnecessary reconsultations [15,16]. Historically, safety-netting processes have been the focus of quality improvement within the cancer clinical and research community, ranging from national strategy documents to local system providers. In health care policy and research, safety-netting has been particularly identified as a tool for facilitating the timely diagnosis of cancer [17-19].

Effective safety-netting results in patient self-care, patients’ recognition of the need for and their prompt seeking of further medical attention, and the timely follow-up of patients [19]. High-quality safety-netting requires clinicians to understand a patient’s information needs, the reasons for safety-netting advice, and the expected clinical course of a condition. Breakdowns in safety-netting communication could occur through the omission of information, by providing information in a way that is not easily understood or remembered, or by failing to address patient concerns [19,20]. Inconsistencies in safety-netting delivery may also harm how advice is perceived and adhered to by patients [9]. Therefore, e–safety-netting tools have a particular role in supporting clinicians’ and patients’ communication, information provision, knowledge, and memory.

## Harnessing the EHR: e–Safety-Netting Tools—How Might They Solve Some of the Problems Above?

EHRs have been mandated for many years in primary care. These systems have been developed to capture clinical information in a way that is clinically relevant and user-friendly. EHR providers regularly update their systems to ensure that users are able to record and retrieve information easily. Over time, EHR systems have built capabilities for supporting wider functionalities, so that clinicians and managers can better support their patient populations. Although safety-netting is embedded into national health care strategies and policies, it is unclear who holds responsibility for it and how it should work [18,21]. Safety-netting is no longer considered solely as a doctor-patient interaction but as a responsibility of the “system,” which should provide robust safety-netting protocols within the EHR [22]. As patients move through the multiple clinical contacts that lead up to a diagnosis, the increased specification of the safety-netting process could reduce the amount of errors in the diagnostic process [2].

e–Safety-netting tools can be integrated into the EHR or be provided by a separate piece of software. Typical functions include, for example, clinician alerts, administrative tasking, templates for standardized codes, tracking dashboards, and additional support (eg, prepopulated referral forms). The tools may support clinicians by tracking patients over a defined time interval, providing templates to guide consultations, or suggesting appropriate referral pathways [23-25]. They may also support patients by sending them trigger text reminders. Using e–safety-netting software in addition to verbal or paper-based safety-netting methods could reduce the amount of diagnostic errors and delays. This could also make improvement easier via the provision of better audit data about safety-netting. The COVID-19 pandemic has driven a surge of new e–safety-netting tools. However, these have not been evaluated by using robust research designs for patient safety interventions [12]. The variations in designs and functions suggest a lack of clarity with regard to how the tools should prevent diagnostic errors and delays.

## What Safety-Netting Failures Could Be Prevented by an e–Safety-Netting Tool?

There is a lack of robust evidence suggesting whether e–safety-netting tools prevent the types of errors that they are designed to prevent. We found 2 evaluation reports of C the Signs (C the Signs Limited)—a software tool for supporting cancer decision-making and management that has been commissioned in various locations in England, United Kingdom. One evaluation found increased cancer detection rates for clinical commissioning groups, who had implemented the tools, when compared to those for groups who had not implemented the tools [26]. However, a second, independent evaluation of C the Signs found that changes to the number of referrals were inconclusive. This report, which was titled *C the Signs evaluation: report for RM Partners* (Frontier Economics, private report received upon request, 2021), found that there was limited evidence of improved cancer detection.

e–Safety-netting tools require substantial further development in order to reach their potential in reducing the amount of diagnostic delays and errors in primary care. In Table 1, we consider the exemplar of an urgent cancer referral pathway (ie, a primary care process in which patients with suspected cancer symptoms are expected to be seen within 2 weeks for further investigations). We give details about the typical errors and outcomes that occur and how e–safety-netting tools may be developed to prevent this [27]. We also indicate whether certain functions have already been developed in prominent e–safety-netting tools that are currently available [23,25,28,29]. Future e–safety-netting tools could explore other potential process errors associated with safety-netting, such as automatically generating an alert if a patient has a number of attendances within a short span of time [30].

**Table 1:** Types of errors that may be mitigated by an e–safety-netting tool. We use the exemplar of an urgent cancer referral pathway.

Setting	Clinical action	Error	Outcome	Role of the e–safety-netting tool	Currently available
Doctor-patient encounter	Primary care physician is unsure whether to refer a patient with abdominal pain to specialist	Physician decides not to investigate further, as they are not aware of clinical guidelines	Delay in investigation or patient referral	Clinical presentation prompts physician to review clinical decision support tool, which reminds primary care physician of the clinical guidelines	Partially
Doctor-patient encounter	Patient visits physician multiple times for the same persistent problem	Physician does not realize that the patient has visited multiple times	Delays in taking action despite a persistent problem	Tool identifies the repeat pattern from coded data and alerts physician	No
After a consultation	Patient with low-risk symptoms is actively monitored	Patient does not reconsult a physician within the expected time frame	Delay in the timely review of symptoms	Tool alerts physician to any delays in the expected reconsultation time frame	Yes
Physician follow-up	Patient is given advice about the need for a suggested investigation	Patient is unclear about the timely review of results or how to obtain results	Delays in taking action after investigation findings	Trigger patient text message regarding reconsulting a physician promptly when results of the investigation are available	Partially
Practice level	Patient is sent to an urgent referral	Patient does not attend the urgent referral	No urgent review by a specialist	Tool identifies nonattendance and sends a message to the patient and primary care physician	Yes
Regional level	Patient is diagnosed with cancer through an emergency pathway	Primary care network does not use this as an opportunity for audit and improvement	Lack of system improvement	Nominated lead for network can review all cancer cases and disseminate learnings	Yes
Patient health record data	Patient with low-risk symptoms presents to primary care physician, resulting in self-care at home	Patient history, including risk factors, is not recorded or visible in health record	Physician is not aware of risk factors in the patient’s history	Alert the physician to the incomplete patient record, including hidden risk factors, during the consultation	No
Patient health record—population	Patient’s clinical risk percentage for a certain condition increases prominently (per the patient’s coded data)	The data are not observed as a whole, and significant patterns are not established	The system does not identify the patient as one requiring further action	Alerts to practice-level team state that clinical risk has reached a specified trigger level for further action (investigations and referrals)	Yes

## Establishing a Framework for What a Good e–Safety-Netting Tool Would Do

e–Safety-netting tool development may be viewed as an extension of EHR tool development. Hitherto, e–safety-netting tools have not been tested with respect to diagnostic safety. There are many frameworks and evidence bases on this topic. We synthesized the relevant parts of 3 publications in particular—(1) the World Health Organization *Technical Series on Safety in Primary Care: Diagnostic Errors*, which addresses how to improve the safety of multiple aspects of diagnostic and administrative work in primary care [31]; (2) Murphy and colleagues’ [32] Safer Dx Trigger Tools Framework, which outlines good practice for the development of electronic tools to improve diagnostic safety; and (3) Vincent and Almaberti’s [33] compendium of safety strategies. Some additional papers and our own knowledge of safety-netting and e–safety-netting tools were used to construct an emergent framework for e–safety-netting tool development (Table 2) [34-36]. This framework may be useful for audits, for e–safety-netting tool development and improvement, and for guiding future research.

**Table 2:** Emergent framework of principles for high-quality e–safety-netting tools.

e–Safety-netting principle	Details	Example
All patients registered will be e–safety-netted.	The tool supports reductions in diagnostic errors for all patients with all types of presentations, not just those who are considered at-risk patients.	The tool has automatic functions that work for all patients (eg, detecting multiple presentations or consultation patterns that might indicate that action is needed and triggering alerts).
All clinicians and primary care staff are responsible for e–safety-netting.	The tool is not reliant on sign-up but is automatically applied for every user registered on the system. The responsibilities would be configured to the users’ credentials (eg, primary care physician, nurse, and receptionist).	The e–safety-netting functions are integrated into the electronic health record and cannot be switched off. Algorithms and alerts are live for every patient.
Limit burden and cognitive bias by using automatic functions, where possible.	The tool functions equally for every patient, not just those selected by the primary care professional or those on a “list.”	Data capture is facilitated by standardized autofill. Patients are automatically selected for follow-up by risk stratification tools.
Support diagnostic processes before, during, and after consultations [34].	The tool supports continuous improvements in data quality and decision-making during the consultation, and it offers memory aids and alerts for both professionals and patients.	The tool notifies primary health care professionals when a patient data record is incomplete. Alerts are triggered or sent to a patient as a reminder to attend an investigation. The physician and patient are alerted when the patient has not attended an investigation, or the physician is alerted when the patient has not attended a specialist appointment.
Monitor, auto-detect, and measure pathway process errors or deviations and alert the relevant people [35].	The tool monitors all appropriate parts of the patient pathway. It automatically detects, rationalizes, and quantifies errors. It also alerts the appropriate staff member to errors of interest.	The tool automatically measures the time interval since the last consultation and agreed upon action. So, if there is delay in presentation, an alert is triggered. If the tool detects that a patient has not fulfilled the prescription, it alerts their health care professional and the patient.
Use simple processes that make it easy to access and transfer complex information.	The tool is easy to navigate, seamless with existing electronic health records, and automatically present at the point of care to support decision-making. Only 1 tool is in use within the primary care system to avoid confusion.	The tool allows for the easy transfer of information to other organizations and has simple and intuitive displays. It also allows users to access up-to-date pathways and referral criteria and has decision support functionalities.
Spread responsibilities and roles within primary care that have an overall impact on the whole patient pathway.	The tool allows the whole clinical and administrative team to use the tool with a centralized alert system, including champions or experts within the team.	There is shared responsibility for “flags” and errors within the system and thus a higher likelihood that the tool will initiate action. The tool supports a culture of shared responsibility.
Support senior leadership to optimize safety strategies within a regular quality improvement program.	The tool creates visual aggregate displays of increased errors (ie, practice dashboards) to establish normative quality standards. It has the ability to self-monitor and self-improve (ie, through artificial intelligence, it improves itself with data and feedback) [11].	The tool allows for the automatic identification of common diagnostic process errors, sends alerts for unexpected increases in error, and has control over the granularity of data.
Allow for patient interaction and feedback [36].	Patients can interact to input either their own health metrics or feedback on symptom changes. Patients can access the appropriate level of information to support themselves in managing their health. Integration with other e-consulting tools is possible.	Patients can self-report attendance to appointments and tick it off. Patients can provide feedback on changes in symptoms to trigger a follow-up appointment. Patients can record and report their weight or blood pressure.

Table 2 outlines 9 principles for e–safety-netting tools that we suggest would denote a high-quality tool. There are currently no tools available that meet all of the criteria in the framework. We hope that the framework will facilitate the development and improvement of e–safety-netting tools. It may also enable national and local audits and analyses, highlighting differences in performance and presenting potential solutions for improvement. Building on the development of new or modified e–safety-netting tools, health system leaders will need to ensure that their organizations have the necessary resources to implement them and to manage and respond to the data generated.

## Discussion

We have presented a framework for structuring the development, evaluation, and implementation of e–safety-netting tools in primary care. The framework includes individual user benefits, technical features, and social aspects of use. Using this framework could support the progress of policies to facilitate the earlier diagnosis of serious diseases, such as cancer, cardiovascular disease, lung disease, diabetes, renal failure, and heart failure [21,37], and increase patient safety [32,38].

The framework is based on principles from established EHR tool development and patient safety frameworks but requires further validation through clinical and public input as well as empirical research. e–Safety-netting development via the use of this framework may require multidisciplinary applied research teams, including software developers; user experience and design; clinical knowledge; applied psychology; health services research; and epidemiological expertise.

The e–safety-netting framework proposed provides an approach to appraising existing tools and guiding e–safety-netting tool development. It would be valuable for commissioners to learn not only from existing experiences of successful adoption but also from decisions to decommission e–safety-netting tools [39]. Currently, there are few opportunities to understand the impact of each available e–safety-netting tool, as they are rarely evaluated and their functions are often updated. Policy makers should make it a condition that these tools be independently evaluated with results that are kept in a centrally held repository [40]. Evaluations would inform local adoption and allow for the alignment of these systems with health care strategies.

Patients need a robust, evidence-based system to ensure that they are monitored until their symptoms have been explained. Without this, primary care services are prone to operational failure. Operational failures (disruptions, errors, or inadequacies in the information, supplies, or equipment needed for patient care) are linked to often time-consuming compensatory actions for ensuring that patient care is coordinated and remains safe. At a time when workloads are continuing to increase in primary care and the format of clinical contacts is changing, e–safety-netting tools offer an approach to distributing the responsibility for follow-up safely among members of practice teams and to patients [41,42]. This is relevant to the development of integrated digital care records and population health management dashboards by integrated care systems [43]. There is further potential to look at the development of e–safety-netting at scale in secondary care and elsewhere [44].

There are likely to be challenges to uptake and implementation, even for tools that conform to the framework we have outlined [45]. However, e–safety-netting tools that align with the individual, social, and technical aspects of primary care work are more likely to succeed [46].

## Abbreviations

EHR: electronic health record
